# Root Membrane Ubiquitinome under Short-Term Osmotic Stress

**DOI:** 10.3390/ijms23041956

**Published:** 2022-02-10

**Authors:** Nathalie Berger, Vincent Demolombe, Sonia Hem, Valérie Rofidal, Laura Steinmann, Gabriel Krouk, Amandine Crabos, Philippe Nacry, Lionel Verdoucq, Véronique Santoni

**Affiliations:** 1 BPMP, CNRS, INRAE, Institut Agro, University Montpellier, 34060 Montpellier, France; nathalie.berger@inrae.fr (N.B.); vincent.demolombe-liozu@inrae.fr (V.D.); sonia.hem@inrae.fr (S.H.); valerie.rofidal@inrae.fr (V.R.); zirkus.laura@web.de (L.S.); gkrouk@gmail.com (G.K.); amandine.crabos@inrae.fr (A.C.); philippe.nacry@inrae.fr (P.N.); lionel.verdoucq@supagro.fr (L.V.); 2 Center for Computational and Theoretical Biology, University of Würzburg, 97070 Würzburg, Germany

**Keywords:** aquaporin, mass spectrometry, osmotic stress, ubiquitination

## Abstract

Osmotic stress can be detrimental to plants, whose survival relies heavily on proteomic plasticity. Protein ubiquitination is a central post-translational modification in osmotic-mediated stress. In this study, we used the K-Ɛ-GG antibody enrichment method integrated with high-resolution mass spectrometry to compile a list of 719 ubiquitinated lysine (K-Ub) residues from 450 Arabidopsis root membrane proteins (58% of which are transmembrane proteins), thereby adding to the database of ubiquitinated substrates in plants. Although no ubiquitin (Ub) motifs could be identified, the presence of acidic residues close to K-Ub was revealed. Our ubiquitinome analysis pointed to a broad role of ubiquitination in the internalization and sorting of cargo proteins. Moreover, the simultaneous proteome and ubiquitinome quantification showed that ubiquitination is mostly not involved in membrane protein degradation in response to short osmotic treatment but that it is putatively involved in protein internalization, as described for the aquaporin PIP2;1. Our in silico analysis of ubiquitinated proteins shows that two E2 Ub-conjugating enzymes, UBC32 and UBC34, putatively target membrane proteins under osmotic stress. Finally, we revealed a positive role for UBC32 and UBC34 in primary root growth under osmotic stress.

## 1. Introduction

Plants are exposed to different types of abiotic stress conditions such as drought or salinity that result in diminished plant growth and crop productivity [1]. Most of these conditions impose osmotic stress on plants by reducing the water potential of the environment. The consequences of osmotic stress manifest as inhibited cell elongation, stomata closure, reduced photosynthetic activity, the translocation of assimilates, changes in various metabolic processes, and disturbances in water and ion uptake. The ability of plants to survive these abiotic stresses relies heavily on their proteomic plasticity. Protein stability, activity, localization, and interactions with partners have all been widely described as being governed by ubiquitination [2,3]. Ub is a 76-amino acid polypeptide that is highly conserved in eukaryotes and is ubiquitously found in tissues. It is linked to either target proteins or itself through the sequential action of three enzyme classes: Ub-activating enzymes (E1s), Ub-conjugating enzymes (E2s), and Ub ligases (E3s) [4]. The activities of these enzymes ultimately result in the covalent attachment of Ub to a lysine (K) residue in the target protein. Ubiquitination can result in the conjugation of a single moiety (mono-ubiquitination), multiple Ub molecules that are individually attached (multi-mono-ubiquitination), or in the form of a chain (poly-ubiquitination) attached to a specific substrate. Poly-Ub chains are formed by the further attachment of Ub moieties linked together by one of the seven lysine residues present in a Ub molecule (K6, K11, K27, K29, K33, K48, and K63), or by the N-terminal methionine in the form of head–tail linear repeats. Poly-Ub chains exhibit different topologies and are associated with diverse biological functions [5]. Poly-ubiquitination involving residue K48 from ubiquitin (K48-Ub linkage) triggers the degradation of target proteins by the 26S proteasome [6], and the K63-Ub linkage includes roles in the endocytosis of plasma membrane proteins, DNA damage responses, and to a lesser extent, autophagy and signaling [7,8].

The Arabidopsis genome contains over 1400 genes encoding E3s, 37 canonical E2s, and 7 E2 variant proteins. The number of E3s associated with abiotic stress and in particular with regulating abscisic acid production, signaling, and response now includes at least 25 different E3s [9]. In addition to E3s, it appears that E2s are not only utilized as Ub-transferring components but also regulate the specificity of target ubiquitination [8,10].

K-Ɛ-GG (DiGly, the remnant from ubiquitinated proteins following trypsin digestion) antibody affinity enrichment provides an efficient method of capturing and concentrating this remnant of ubiquitinated proteins treated with trypsin prior to MS/MS [11,12]. Here, we used such a technology coupled to deep MS/MS analysis of Arabidopsis root proteins, before and after a short-term osmotic treatment of plants. We provide an extensive inventory of K-Ub residues in a membrane protein fraction. We show a role for ubiquitination outside of the membrane protein degradation process in response to short-term osmotic treatment and pinpoint two E2 Ub-conjugating enzymes, UBC32 and UBC34, as positive regulators of primary root growth during osmotic stress.

## 2. Results

**Figure 1:**
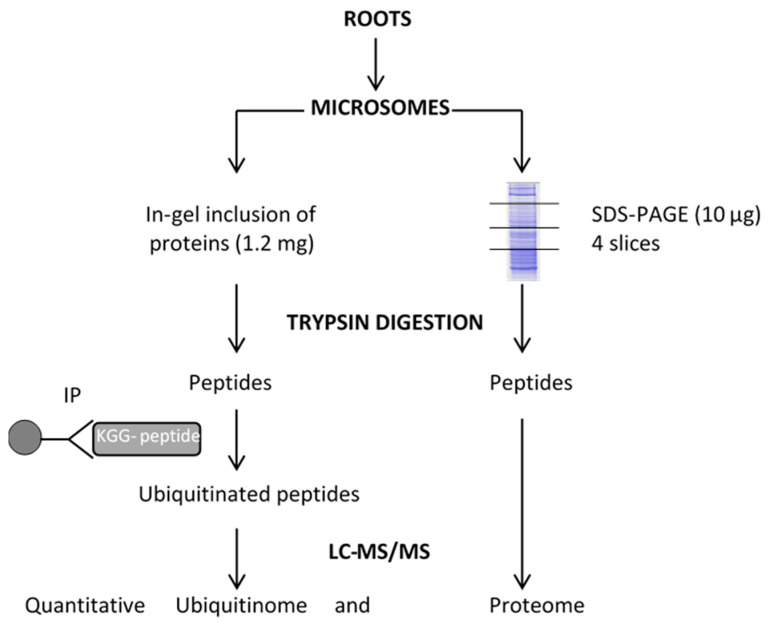
Workflow for quantitative profiling of the proteome and the ubiquitinome in Arabidopsis root membrane proteins upon mannitol treatment. LC-MS/MS: liquid chromatography–tandem mass spectrometry. IP: immunopurification.

The ubiquitinome response to osmotic stress was investigated by treating plants with 200 mM mannitol for 1 h, followed by a combined quantitative analysis of the proteome and ubiquitinome of a microsomal fraction, according to the proteomic workflow described in Figure 1.

### 2.1. Differentially Accumulated Proteins in Response to Mannitol Treatment

A total of 6081 proteins were identified based on identification with at least two peptides (Table S1), of which 26% were transmembrane proteins (Table S1). Using gene ontology (GO) analysis of cell component terms, we showed that a majority of GO terms were associated with membrane proteins, even when the extrinsic proteome (i.e., proteins without any transmembrane domain) was exclusively considered (Figure S1). These results show that the microsomal fraction is enriched in membrane proteins. Treating plants with 200 mM mannitol for 1 h resulted in 226 differentially accumulated proteins (DAPs): 132 were up-accumulated (average increase: 1.51×), and 94 were down-accumulated (average decrease: 0.61×) (Table S2). In addition, 1 protein appeared upon mannitol treatment, while 10 proteins disappeared (Table S2). DAPs were classified according to the GO functional categories of “biological process”, “molecular function”, and “cellular component” (Figure S2). Interestingly, enriched GO terms mostly concerned ATPase activities (Figure S2), in agreement with observations showing the tight regulation of plasma membrane H^+^-ATPase in response to several biotic and abiotic stress responses [13].

### 2.2. Characterization of the Root Membrane Ubiquitinome

**Figure 2:**
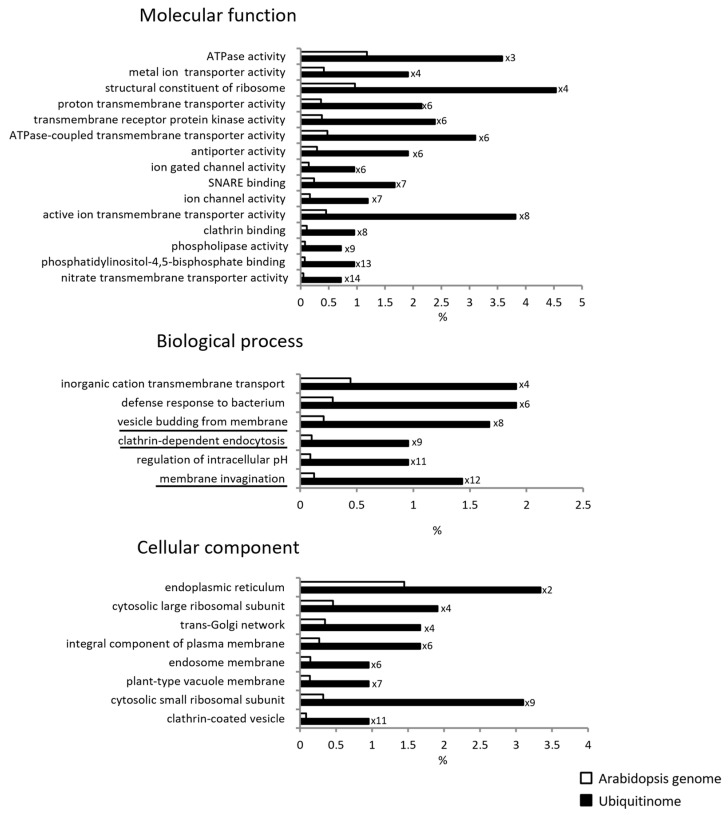
Functional enrichment analysis of ubiquitinated root microsomal proteins. The percentage is calculated with regard to the number of identified ubiquitinated proteins (black) and the total number of Arabidopsis proteins (white). Numbers indicate the fold enrichment by comparison with the Arabidopsis genome. Underlined biological processes concern proteins involved in intracellular trafficking and include 15 genes (Table S3).

To identify ubiquitinated proteins in Arabidopsis roots, we combined immunoaffinity enrichment (using a high quality anti-K-Ɛ-GG antibody; PTM biolabs) and high-resolution mass spectrometry. A total of 719 ubiquitinated peptides harboring a total of 786 K-Ub residues belonging to 450 proteins were identified, 264 of which contained at least one transmembrane domain (Tables S3 and S4). Our GO enrichment analysis showed that ubiquitinated proteins were enriched in transporters, in proteins involved in the regulation of intracellular pH, and in cellular trafficking processes that were characterized by the GO terms “vesicle budding from membrane”, “clathrin-dependent endocytosis”, and “membrane invagination” (Figure 2) and included 15 proteins (Table S3). These observations suggest that membrane proteins, as well as the proteins that drive their trafficking are ubiquitinated.

**Figure 3:**
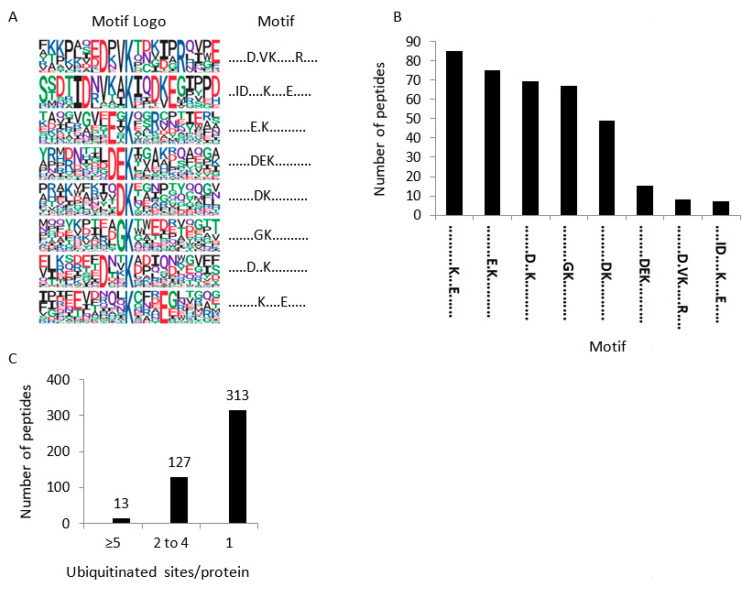
Motif analysis of identified K-Ub residues in root microsomes. (**A**) Ubiquitination motifs and the conservation of K-Ub residues. The height of each letter corresponds to the frequency of the amino acid residue in that position. The central K refers to the K-Ub residue. (**B**) The number of identified peptides containing a K-Ub residue in each motif. (**C**) The number of K-Ub sites per protein.

Consensus peptide motifs for K-Ub residues were extracted using p-logo [14]. In total, 643 unique ubiquitinated sites were unable to highlight one unique motif (Table S5, Figure 3A,B). However, the presence of an acidic amino acid close to K-Ub was observed in all motifs except one. We next examined ubiquitinated peptides arising from the Ub protein itself to gain insight about poly-Ub linkages that occur in a protein sample. Footprints on each of the seven internal lysines were identified (K6, K11, K27, K29, K33, K48, K63) (Figure S3), indicating multiple Ub-linkages within membrane proteins. Although peptide intensity is not indicative of the absolute quantity of each Ubi-peptide, the K48- and K63-Ub linkages appeared to predominate the poly-Ub linkages (Figure S3). Most of all, our results reveal that mannitol treatment does not significantly modify the proportion of each poly-Ub linkage (Figure S3).

### 2.3. Differentially Accumulated Ubiquitinated Proteins in Response to Mannitol Treatment

Out of 374 quantified ubiquitinated peptides, 82 showed quantitative variations in which 54 ubiquitinated peptides were up-accumulated and 28 were down-accumulated (Table S6). Enrichment-based clustering analyses showed that the ubiquitination of proteins altered by mannitol treatment mainly concerns ATPase, transporters, and SNARE binding activities (Figure S4). An inverse quantitative relationship between a protein’s abundance and its ubiquitinated form could be indicative of a role for ubiquitination in protein degradation. However, none of the 43 up-accumulated ubiquitinated peptides were affiliated with a decreased abundance in the corresponding protein (Table 1). In addition, among 24 down-accumulated ubiquitinated peptides, only 6 of them corresponded to accumulated proteins, including CARK1, HIR2, NRT2;1, PIRL5, At1g48210.2, and At3g47210.1 (Table 1). Thus, a role for ubiquitination in degrading these proteins could be considered. By contrast, for a majority of the proteins, the absence of any inverse quantitative relationship between the protein and its ubiquitinated form suggests that upon short-term osmotic treatment, ubiquitination could interfere with protein function or cellular localization rather than with protein stability.

### 2.4. Ubiquitination of PIP Aquaporins

Aquaporins define a large family of ubiquitous integral membrane proteins that mediate the transport of water and small neutral solutes across membranes [18]. Thirty-five homologs within four homology subclasses have been identified in Arabidopsis. The plasma membrane intrinsic proteins (PIPs) consist of 13 isoforms further subdivided into the PIP1 and PIP2 subgroups that were all identified in this study (Table S1). Nine of them exhibited ubiquitinated residues in their N- and/or C-terminus (Table S3, Figure S5). Since only three lysine residues are described in the literature as being ubiquitinated [11,17], the present work greatly increases our knowledge regarding the ubiquitination of PIPs. Increased ubiquitination of K3 and K276 was observed in PIP2;1 upon short-term mannitol treatment (Table 1). K276 ubiquitination was recently shown to mediate PIP2;1 degradation upon long-term drought [17]. However, since we observed that PIP2;1 cellular abundance was unchanged (Table 1), the increased ubiquitination of K276 observed when plants are subjected to a 1 h mannitol treatment cannot be involved in PIP2;1 degradation.

**Figure 4:**
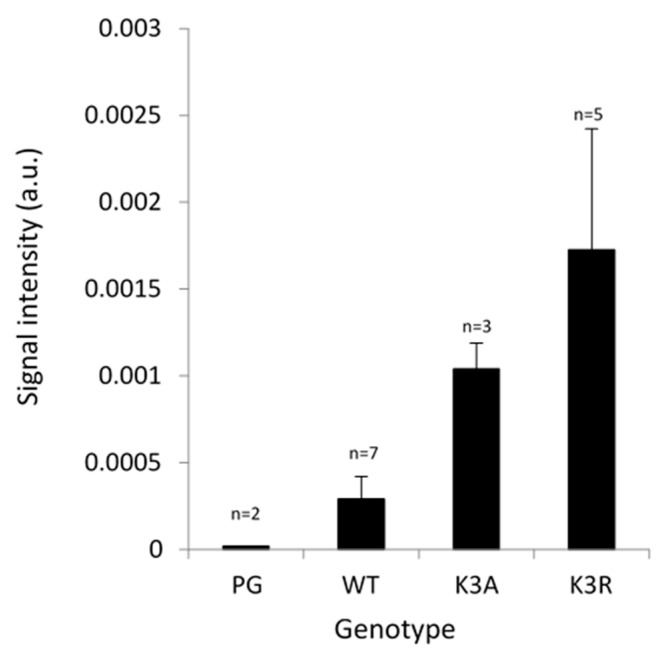
Relative abundance of PIP2 isoforms in Arabidopsis suspension cells overexpressing PIP2;1 WT and carrying a point mutation of K3 in alanine (K3A) and in arginine (K3R). ELISA assays were performed with total protein extracts and anti-PIP2 antibody [19]. The number of independent stable cell lines is indicated. Data were from three individual ELISA assays per cell line. Standard error is shown.

Next, we checked whether there is a role for K3 ubiquitination using a simplified system of Arabidopsis suspension cells with a low basal level of endogenous PIPs. We overexpressed PIP2;1 either wild-type or carrying point mutations at K3 in alanine (K3A) and in arginine (K3R) [19] with the aim of preventing ubiquitination at that site. We previously showed by Western blot analysis of total protein extracts using an anti-PIP2;1 peptide antibody that there is a significantly strong overexpression of PIP2;1 in these cells, as compared with untransformed cells or cells transformed with an empty vector (PG) [19]. Here, using an ELISA assay, we observed a significant accumulation of PIP2;1 in suspension cells overexpressing PIP2;1-K3A and PIP2;1-K3R as compared with PIP2;1-WT (Figure 4), suggesting that K3 could be a ubiquitinable residue participating in the degradation of PIP2;1. However, in roots placed under short-term osmotic treatment, increased K3 ubiquitination did not correlate with decreased PIP2;1 abundance, suggesting (as for K276) an additional role for K3 ubiquitination outside of PIP2;1 degradation.

### 2.5. The Interactome of Ubiquitinated Proteins

**Figure 5:**
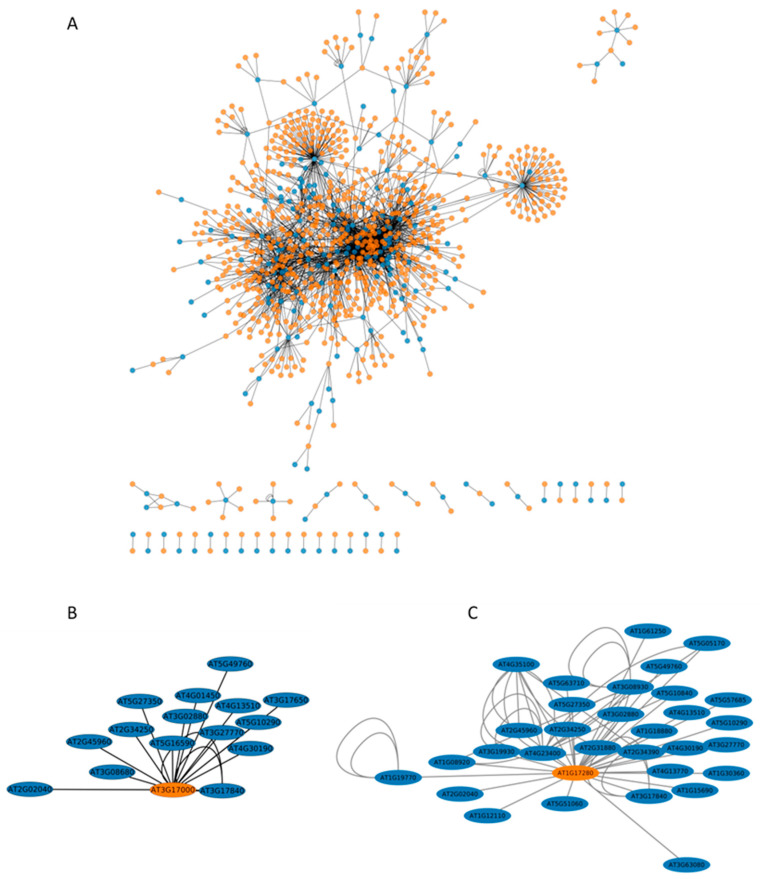
The interaction network of ubiquitinated proteins. Interactants from a Y2H approach [15] and Split-Ub approach [16,20] were considered, and the network was visualized by Cytoscape (version 3.7.2). (**A**) The network includes ubiquitinated proteins (blue) together with their reported interactants (orange) (details in Table S7a). (**B**) The UBC32 subnetwork (details in Table S7b). (**C**) The UBC34 subnetwork (details in Table S7c).

For additional insight into the extent of the role of ubiquitination, we next constructed a network for the ubiquitinated proteins identified in this work and their interactants identified in previous yeast-two hybrid [15] and Split-Ub global approaches [16,20]. This final network consisted of 1011 proteins (Table S7a, Figure 5). Transport and trafficking functions were enriched in such interactome, and interestingly, the most enriched process concerns ubiquitination, with the GO term “protein K63 linked ubiquitination” showing a 37-fold enrichment (Figure S6). Five E2s (UBC26, UBC32, UBC34, UBC35, UEVD1) and one E3 (UPL6) were identified in this network (Table S7a). We investigated the extent of interaction of these enzymes with the ubiquitinated proteins identified in the present work and identified two E2s, UBC32 and UBC34, which putatively interact with 15 and 31 ubiquitinated transmembrane proteins involved in water and ion transport, respectively (Figure 5, Table S7b,c).

A similar approach that focused specifically on DAUPs and their interactants (Table S7d, Figures S7 and S8) also identified UBC32 and UBC34 as E2s targeting nine proteins whose ubiquitination changed with mannitol treatment (Figure S7, Table 1). These proteins include NRT1;1-PTR8.3, NRT1-PTR6.3, two LRR-Receptor like kinases, a nodulin MtN21-like transporter, AHA2, the secretory carrier-associated membrane protein SCAMP3, the peroxidase GPX5, and an unknown protein.

### 2.6. The Osmotic Phenotype of Ubc32, Ubc33 and Ubc34 Mutants

**Figure 6:**
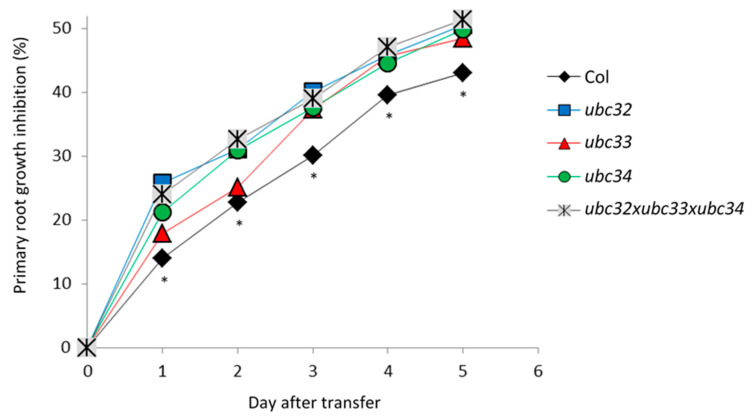
Inhibition of primary root length by mannitol in Arabidopsis thaliana WT plants (Col) and *ubc32*, *ubc33*, *ubc34*, and *ubc32xub33xubc34* mutants. Fifteen plants per condition were grown for 5 days in MS medium and then transferred to MS medium and MS medium supplemented with 200 mM mannitol. Primary root length was monitored up to 5 days after transfer (Figure S9). Asterisks (*) mean that the WT is significantly different from at least one mutant in a one-way ANOVA test combined with a Tukey test (*p*-value between 0.01 and 0.001 (statistics in Table S8)).

Root responses to osmotic stress involve high plasticity in root growth and architecture, which is partly determined by primary root growth. To reinforce the roles of UBC32 and UBC34 in the adaptive root response to osmotic stress, we studied the primary root growth phenotype of corresponding mutants under control conditions and upon osmotic treatment. Since AtUBC32 and AtUBC34 belong to a small gene subfamily including AtUBC33 [21], we also studied the *ubc33* mutant and a triple knockout mutant line, *ubc32xubc33xubc34*, due to the putative redundancy between the three genes. Five-day-old plants were transferred to either control MS medium or MS medium supplemented with 0.2 M mannitol, and primary root length was monitored for up to 5 days after transfer (Figure 6 and Figure S9, Table S8). The primary root growth in control WT plants (Col) was inhibited by 14% one day after transfer and by up to 43% 5 days later (Figure 6). The *ubc32* and *ubc34* single mutants and the triple mutant showed a significantly higher primary root growth inhibition ranging from 25% at day 1 after transfer up to 50% at day 5 after transfer (Figure 6). Thus, suppression of AtUBC32 and AtUBC34 favored primary root growth inhibition under mannitol treatment, suggesting that these genes contribute to primary root growth under osmotic stress. This result is observable after only one day of treatment, suggesting that these genes play an early role in the response to osmotic stress.

## 3. Discussion

### 3.1. A Resource of Ubiquitinated Membrane Proteins

Our study included 450 ubiquitinated proteins, significantly increasing the database size of ubiquitinated membrane protein substrates in plants. In comparison with other large-scale Arabidopsis ubiquitinomes [8,11,22,23,24,25,26,27], 207 novel proteins, 90% of which are transmembrane proteins, were identified as being ubiquitinated (Table S9). We also observed that 20% of ubiquitinated peptides can be additionally modified by oxidation, phosphorylation, and acetylation (Table 1 and Table S4), adding complexity to cellular signaling. The major proton pump AHA2 harbors 21 K-Ub sites (Table S3), 4 of them showing quantitative variations upon mannitol treatment (Table 1). K888 in particular is ubiquitinated, and this site is located close to T881, whose phosphorylation leads to pump activation [28]. Moreover, the doubly modified peptide (i.e., by phosphorylation and ubiquitination) was accumulated upon mannitol treatment. Recent studies have highlighted the importance of crosstalk between phosphorylation and ubiquitination in several plant signaling pathways [29], and the presence of such a K-Ub near a critical phospho-residue questions the role of this ubiquitination in the modulation of ATPase function.

Even though no ubiquitin motif could be described, the K-Ub residue appeared to be preferentially surrounded by an acidic residue (Figure 3). Similar observations can be obtained from ubiquitinome studies in petunia flower and rice embryo [12,30]. By contrast, previous studies in rice leaf, wheat seedling, and maize leaf have reported that alanine is enriched around K-Ub [31,32,33]. Thus, although no Ub motif could be identified, the presence of an acidic residue appears to be a common feature between three different tissues (root, flower, embryo), whereas the close presence of an alanine residue appears to be more specific for ubiquitinated residues in leaf proteins.

In two recent studies, a total of 422 proteins were identified as carrying a K63-Ub linkage [8,34]. A total of 74 of these proteins were identified in the present study, 29 of which were identified as carrying a unique K-Ub site that can be preferentially accounted for a K63-Ub linkage (Table S10). However, 44 proteins were also described in the present study as harboring at least two K-Ub sites (Table S10). This suggests that either all K-Ub residues are involved in K63-Ub linkage or that different types of Ub linkages coexist within the same protein, including K63-Ub. This type of coexistence has already been described in animals [35,36] and more recently in plants: the ubiquitination pattern of oleosin includes K48-Ub linkage that induces the proteasomal protein degradation, as well K63-Ub linkage, the role of which remains unknown [37]. Thus, the specific fate of proteins can be dictated by specific coexisting poly-Ub linkages.

In addition to transporters and channels, we reported that vesicle transport-related proteins including clathrin assembly proteins, the AP-2 complex subunit, v-SNARE proteins, vesicle-associated proteins, and syntaxins were overrepresented in the ubiquitinome (Table S3). Endocytosis and endosomal trafficking are essential processes in cells for controlling the dynamics and turnover of plasma membrane proteins [38]. The recruitment of cargo into endocytic vesicles (e.g., clathrin-coated pits) involves the ‘endosomal sorting complex required for transport’ (ESCRT) multi-subunit complex and requires adaptor proteins to eventually associate with clathrin [38]. The vesicles fuse with the acceptor organelle in a process mediated by factors such as SNAREs and small GTPases [38]. Here, we unexpectedly observed a concomitant ubiquitination of cargos and proteins from the endocytic machinery (Table S3). While a role for the ubiquitination of cargos in their endocytosis has recently emerged in plants [39], the ubiquitination of proteins involved in endocytosis is rarely documented. In animals, several proteins involved in epidermal growth factor (EGF) receptor endocytosis were shown to be ubiquitinated after EGF stimulation [40]. In yeast, ubiquitination was recently shown to function as a recycling signal for sorting a SNARE into COPI vesicles in a non-degradative pathway [41]. Therefore, our results suggest a role for ubiquitination in regulating the function of proteins involved in endocytic trafficking, highlighting a broad role for ubiquitin in internalizing and sorting cargo proteins.

### 3.2. The Role of Ubiquitination in Response to Short-Term Osmotic Treatment

Because ubiquitination can induce protein degradation, we looked for an inverse relationship between the abundance of proteins and their ubiquitinated form that could indicate a role for ubiquitination in protein degradation. Caution must be taken with this assumption since it is not until the ubiquitin chain is assembled that it may act as a degradation signal [42]. If a large proportion of ubiquitin is likely to be attached as mono-ubiquitin, this might skew the inverse relationship between the abundance of a protein and its ubiquitinated form. Although we may have overestimated the number of concerned proteins, only 10% of them (n = 6) exhibited this inverse relationship, whereas 90% showed ubiquitination changes without any change in protein abundance (Table 1). Thus, for a majority of these membrane proteins, ubiquitination is not involved in protein degradation in response to short-term osmotic treatment. In particular, PIP2;1 abundance was unchanged upon short-term mannitol treatment, while its ubiquitination increased at K3 and K276 (Table 1). This osmotic treatment was shown to induce maximal root hydraulic conductivity inhibition by 60%, which can be accounted for by a decrease in aquaporin function [43] and not PIP degradation since the abundance of all PIPs was unchanged (Table S2). Thus, even though ubiquitination at K3 and K276 can be involved in PIP2;1 degradation (Figure 5, [17]), we assume that ubiquitination induces different consequences upon short-term mannitol treatment. Indeed, short-term osmotic treatment induces PIP2;1 selective endocytosis [44]. In addition, PIP2;1 is ubiquitinated by K63-Ub linkage [34], a poly-Ub linkage that plays a general function in the sorting of endocytosed cargos by the endosomal sorting complex required for transport [39]. Thus, we hypothesize that the increase in PIP2;1 ubiquitination induced by short-term treatment should participate in internalizing PIP2;1 and not in degradation of the protein. This result contrasts with the PIP2;1 degradation observed under long-term drought treatment, due to the simultaneous activity of UBC32 and the E3 ligase Rma1 in ubiquitinating PIP2;1 at K276 [17]. The pairing of E3s with different E2s is dynamic and changes in response to external stimuli [10]. Thus, under short-term osmotic stress, a specific E2/E3 combination that differs from UBC32/Rma1 could regulate PIP2;1 internalization. However, this hypothesis will require additional experiments.

### 3.3. UBC32 and UBC34 Contribute to Primary Root Growth under Osmotic Stress

E2s have recently emerged as key mediators of chain assembly, in particular by dictating the K residue within Ubused to link the moieties in a chain [10]. Our protein–protein network analysis identified UBC32 and UBC34 as E2s that putatively interact with proteins whose ubiquitination changed upon short mannitol treatment (Table 1, Figure S7). Recent expression studies have suggested that E2s participate in abiotic stress responses [45,46,47]. Surprisingly, the role of UBC32 appears contradictory since it has been described as playing both negative and positive roles in response to long-term drought in [21] and [17], respectively. Our study reveals that, in the context of short-term osmotic treatment, UBC32 and UBC34 positively regulate primary root growth, thus playing a positive role in osmotic stress tolerance. UBC32, UBC33, and UBC34 are all reported to participate in the endoplasmic reticulum-associated degradation (ERAD) pathway in Arabidopsis, which is a major degradation system involved in removing misfolded or unfolded proteins retained in the ER [17,48,49,50,51,52]. The involvement of ERAD components suggests that short-term osmotic stress may also result in ER/protein stress, which engages ERAD to control the secretion of plasma membrane proteins. However, upon 1 h mannitol treatment, proteins that putatively interact with UBC32 and UBC34 and show increased ubiquitination did not display any decreased cellular abundance (Table 1). We thus speculate that UBC32 and UBC34 are not simply ERAD components but that they also participate in the ubiquitination process in other subcellular organelles such as the plasma membrane. In particular, an internalization of the aquaporin PIP2;1 [17] was observed upon a short-term osmotic stress [44]. We thus speculate that, upon a short-term osmotic stress, a rapid coordinated internalization of aquaporins and of ions transporters involved in plant mineral nutrition could contribute to maintain a minimal root growth. Conversely, absence of internalization would impair root development. Soil is extremely heterogeneous, and root growth maintenance under unfavorable local environment could allow the root tip to reach a more favorable environment and then to maintain longer-term root foraging and plant survival.

## 4. Materials and Methods

### 4.1. Plant Materials and Growth Conditions

Arabidopsis thaliana ecotype Columbia (Col-8) was used as the control wild-type (WT) plant. The *atubc32* (SALK_092817), *atubc33* (GABI_105_D10), *atubc34* (SAIL_1249_C08) single T-DNA insertion mutant alleles and the triple mutant *atubc32xatubc33xatubc34* were obtained from [35]. Homozygosity of these mutants was verified using primers described in [21]. For proteome and ubiquitinome studies, WT seeds were surface-sterilized, sown in 0.2 mL tubes containing 0.8% agar prepared in half strength Hoagland-based solution buffered with MES at pH 5.7 (0.5 mM KH_2_PO_4_, 1.25 mM KNO_3_, 0.75 mM MgSO_4_, 1.5 mM CaNO3, 50 µM H_3_BO_3_, 0.7 µM CuSO_4_, 1 µM ZnSO_4_, 12 µM MnSO_4_, 0.24 µM Na_2_MoO_4_, 50 µM Fe^3+-^EDTA) and vernalized. After 7 days in the growth chamber, the bottoms of the tubes were cut off prior to transfer in 2.5 L opaque recipients in the same medium. Plants were grown for 8 weeks under short-day conditions (8 h/16 h day/night; 23 °C/20 °C day/night) at a light intensity of 160 µmol·m^−2^·s^−1^ and 65% humidity. Plants were treated with 0.2 M mannitol for 1 h. Roots were harvested and stored at −80 °C until analysis.

### 4.2. Microsome Extraction

Roots were crushed with a PULVERISETTE 2 Mortar Grinder (Fritsch) in liquid nitrogen and microsomal proteins were extracted according to [53], except that the grinding buffer contained 10 mM N-ethylmaleimide and that the pellets were resuspended with a potter in 200 µL of Laemmli buffer (65 mM Tris–HCl, pH 7.5, 5% glycerol, 2% SDS) [53]. Proteins were quantified using a detergent compatible with the Bradford assay kit (Thermo Scientific, Waltham, MA, USA).

### 4.3. Protein Digestion

For proteome analysis, three independent biological replicates from the control condition and mannitol-treated plants were used. An amount of 10 µg of microsomes was fractionated using 10% precast SDS-PAGE gel electrophoresis (Bio-Rad, Hercules, CA, USA). After staining with Coomassie blue (R250, Bio-Rad), the gel was rinsed with acetic acid/methanol (Destain, Bio-Rad). Each lane was cut into 4 bands. For the ubiquitinome study, 2 independent biological replicates from the control condition and mannitol-treated plants were used, and microsomal fractions (1.2 mg) were subjected to an in-tube acrylamide inclusion (13% acrylamide/bis-acrylamide, 0.6% ammonium persulfate, 2.5% TEMED) adapted from [54]. For proteome and ubiquitinome analyses, gel slices were treated according to [55], with the exception that proteins were alkylated with 50 mM chloroacetamide for ubiquitinome experiments. Proteins were digested with trypsin (Sequencing Grade Modified Trypsin, Promega, Madison, WI, USA) at a 1:50 (trypsin/protein) ratio at 37 °C overnight. Peptides were extracted according to [55].

### 4.4. Enrichment of Ubiquitinated Peptides

Tryptic peptides were filtered through a C18 cartridge (Sep-Pack Classic, Waters) equilibrated with 0.1% TFA. After loading on the column, the peptides were washed twice with 0.1% TFA and then with 0.1% TFA and 5% ACN. Peptides were eluted with 0.1% TFA and 40% ACN, pooled, frozen overnight at −80 °C, and finally evaporated. Immunoprecipitation experiments were performed with 15 µL of Pan anti-glycine lysine antibody conjugated to agarose beads (PTM Biolabs, Chicago, IL, USA) according to the manufacturer’s instructions. Briefly, tryptic peptides were dissolved in 300 µL of WASH I (100 mM NaCl, 1 mM EDTA, 20 mM Tris-HCl, 0.25% n-Dodecyl β-D-maltoside, pH 8.0), incubated 4 h at room temperature on a rotary shaker, and then sequentially washed 3 times with WASH I, 3 times with WASH II (100 mM NaCl, 1 mM EDTA, 20 mM Tris-HCl, pH 8.0), and 3 times with HPLC-grade water. The elution was performed 3 times with 100 µL of 0.1% TFA. For the LC-MS/MS experiment, 300 µL of pooled eluates was dried under vacuum centrifuge and resuspended in 2% FA.

### 4.5. LC-MS/MS Analysis

The LC-MS/MS experiments were performed using a NCS 3500RS-ProFlow nano system (Thermo Fisher Scientific Inc., Waltham, MA, USA) interfaced online with a nano easy ion source and a Q-Exactive Plus Orbitrap mass spectrometer (Thermo Fisher Scientific Inc, Waltham, MA, USA). The samples were analyzed in a data-dependent acquisition mode. For total proteome and ubiquitinome experiments, 2 µL and 6 µL of peptides were injected, respectively. Peptides were first loaded onto a pre-column (Thermo Scientific PepMap 100 C18, 5 µm particle size, 100 Å pore size, 300 µm i.d. ×5 mm length) from the Ultimate 3000 autosampler with 0.05% TFA in water at a flow rate of 10 µL/min. The peptides were separated by reverse-phase column (Thermo Scientific PepMap C18, 3 μm particle size, 100 Å pore size, 75 μm i.d. ×50 cm length) at a flow rate of 300 nL/min. After a 3 min loading period, the column valve was switched to allow elution of peptides from the pre-column onto the analytical column. The loading buffer (solvent A) consisted of 0.1% FA in water, and the elution buffer (solvent B) was 0.1% FA in 80% ACN. The employed 3-step gradient consisted of 4–25% of solvent B until 50 min for ubiquitinome (103 min for total proteome), then 25–40% of solvent B from 50 to 60 min for ubiquitinome (from 103 to 123 min for total proteome), and finally 40–90% of solvent B from 60 to 62 min (123 to 125 min for total proteome). The total run time was 90 min for ubiquitinome (150 min for total proteome), including a high organic wash step and a re-equilibration step. Peptides were transferred to the gaseous phase with positive ion electrospray ionization at 1.8 kV. In the data-dependent acquisition procedure, the top 10 precursors were acquired between 375 and 1500 m/z with a 2 Th (Thomson) selection window, a dynamic exclusion of 40 s, a normalized collision energy of 27, and resolutions of 70,000 for MS and 17,500 for MS2. Spectra were recorded with Xcalibur software (4.3.31.9) (Thermo Fisher Scientific). The mass spectrometry proteomics data were deposited at the ProteomeXchange Consortium via the PRIDE partner repository with the dataset identifier PXD022249.

### 4.6. Identification and Quantification of Whole Proteome and Ubiquitinome

For the proteome and the ubiquitinome, the resulting MS/MS data were processed using MaxQuant with an integrated Andromeda search engine (version 1.6.6.0). Tandem mass spectra were searched against the TAIR10 database (35,417 entries). The minimal peptide length was set to 6. The criteria “Trypsin/P” was chosen as the digestion enzyme. Carbamidomethylation of cysteine was selected as fixed modification and methionine oxidation, N-terminal acetylation, and phosphorylation (S/T/Y) were systematically selected as variable modifications. Up to 4 missed cleavages were systematically allowed.

For proteome analysis, the mass tolerance of the precursor was 20 and 4.5 ppm for the first and main searches, respectively, and was 20 ppm for the fragment ions. The function “match between run” was used. Proteins were identified provided that they contained one unique trypsin peptide. The rates of false peptide sequence assignment and false protein identification were fixed to be lower than 1%. Quantification was performed with at least 2 peptides per protein, 1 of them unique to the protein. To investigate differentially expressed proteins, Student’s t-test was performed using protein Label-Free Quantification intensity values when present in at least 2 replicates and in at least 2 biological replicates per condition.

For ubiquitinome analysis, “GlyGly” on K residue was additionally specified as a variable modification. Ubiquitinated peptides were considered as long as they had a score >40 and if they were identified in at least two independent samples. The function “match between run” was not applied. The intensity of each peptide from the “evidence” table was normalized to the sum of all peptide intensities in each sample, and a *t*-test was performed to investigate differentially expressed peptides. The ubiquitinated peptides with consistent fold changes in two replicates were counted, and the significance of the abundance change among samples was evaluated as differentially expressed by a Student’s *t*-test. A *p*-value < 0.05 was considered statistically significant. The appearance/disappearance of peptides was considered on condition of their presence in two biological replicates and the corresponding absence from the two other biological replicates. We defined “absence” as no razor or unique peptide in any biological condition replicate and “presence” as the identification of at least one unique peptide in all replicates of a biological condition.

### 4.7. Bioinformatic Analyses

GO term association and enrichment analyses were performed using Panther (http://www.pantherdb.org/, version 16.0, accessed on 1 April 2021) [56]. Fold enrichments were calculated based on the frequency of proteins annotated to the term compared with their frequency in the Arabidopsis proteome. The *p*-value combined with the false discovery rate correction was used as criteria of significant enrichment for GO catalogs, whereas a *p*-value < 0.05 was considered to be enriched for GO terms. The most specific subclasses were considered. The GO annotation was classified based on the “biological processes”, “molecular functions”, and “cellular components” categories. GO terms were reduced with rrvgo (https://ssayols.github.io/rrvgo/, version 1.5.4, accessed on 5 April 2021). The number of transmembrane domains was estimated with Aramemnon (http://aramemnon.botanik.uni-koeln.de/, version 8.1, FlüggeLab, Cologne, Germany, accessed on 3 June 2021). The p-logo software [14] (https://plogo.uconn.edu/, UCONN, university of Connecticut, USA, accessed on 9 June 2020) was used to analyze the models of the sequences with amino acids in specific positions of ubiquitin-21-mers (10 amino acids upstream and downstream of the K-Ub site) in all of the protein sequences. In addition, the Arabidopsis proteome was used as the background database, and the other parameters were set to default values. Protein–protein interaction data were obtained from plant interactome databases, including results from a yeast two-hybrid approach [15] and from Split-ubiquitin approaches [16,20], in order to build a network including these ubiquitinated proteins together with their reported interactants. Protein–protein interaction networks were visualized using Cytoscape version 3.7.2 [57].

### 4.8. Ectopic Expression of PIP2;1 in Suspension Cells

Mutated PIP2;1 cDNAs were constructed according to [19]. Biolistic transformation of 5-day-old suspension cells was performed as described in [19], and transformed cells were selected on 50 mg/L of hygromycin. Briefly, independent transformed cells were isolated and further cultured on 40 mg/L of hygromycin. Stable insertion of the T-DNA was checked by PCR, and the expression of PIP was detected by Western blot, as described in [19]. Extraction of total proteins from suspension cells and ELISA measurements of PIP2 abundance were performed as described in [19].

### 4.9. Root Architecture Analyses

Plants were stratified for 2 days at 4 °C and grown vertically on agar plates containing half-strength Murashige and Skoog (MS) medium supplemented with 1% (*w*/*v*) sucrose and 2.5 mM MES-KOH pH 6, in a self-contained imaging unit equipped with a 16 M pixel linear camera, a telecentric objective, and collimated LED backlight. Plants were grown in the imaging automat dedicated growth chamber at 23 °C in a 16 h light/8 h dark cycle with 70% relative humidity and a light intensity of 185 µmol·m^−2^·s^−1^ (Vegeled Floodlight, Colasse Seraing, Belgium). Plates were imaged every day for 5 days.

## 5. Conclusions

Our data present the ubiquitinome of root membrane proteins and its variation under osmotic stress. Importantly, the results highlight specific post-translational modification patterns and suggest approaches for exploring the physiological role of lysine ubiquitination in plants under osmotic stress. Our results open new perspectives in the involvement of ubiquitination and trafficking of root plasma membrane transporters in response to changes in local environment, and future studies exploring the function of these ubiquitination in PIP2;1 and ion transporters will be necessary to delineate its role in the root nutrient foraging.

## Figures and Tables

**Table 1:** Variations in the ubiquitinated peptide and the corresponding protein in response to mannitol treatment.

AGI	Description	Ubi-Peptide Sequence	K-Ub	Ubi- Peptide Ratio	Protein Ratio	UBC 32	UBC 34
**proteins with decreased ubiquitination**							
AT1G01580.1	FRO2	IEAFITRDNDAGDEAKAGK	528	DISP	INV		
AT1G08090.1	NRT2;1	ATLEKAGEVAKDKFGK	259	DISP	1.92 (*p* = 0.03)		
AT1G13480.1	Protein of unknown function	LDSELTSLGKSIEIGK	211	DISP	INV		
AT1G32450.1	NRT1;5	S_ac_CLEIYNKDTM_ox_KK	9 or 13 or 14	DISP	INV		
AT1G48210.2	Protein kinase superfamily protein	LSEDKVKQCVDAR	300	DISP	1.22 (*p* = 0.03)		
AT1G58030.1	CAT2	DGLLPSIFSDINKR	369	DISP	INV		
AT2G17440.1	PIRL5, ras group-related	DITEKGAQAVVQYMNDLVEAR	484	DISP	1.51 (*p* = 0.01)		
AT2G23200.1	receptor-like protein kinase	SKGTIDEILDPSLIGQIETNSLKK	710	DISP	INV		
AT2G31610.1	40S ribosomal protein S3-1	TQNVLGEKGRR	62	0.33 (*p* = 0.01)	INV		
AT3G01290.1	HIR2	AEGEAESKYLSGLGIAR	196	DISP	1.23 (*p* = 0.02)		
AT3G04840.1	40S ribosomal protein S3a-1	NVGKTLVSR	45	DISP	INV		
AT3G17410.1	CARK1	LSEDKVKQCVDAR	301	DISP	1.27 (*p* = 0.03)		
AT3G47210.1	Protein of unknown function	KYIISYINEQVELDSR	62	DISP	1.28 (*p* = 0.03)		
AT3G51550.1	Feronia	VLGVGGFGKVYR	549	DISP	INV		
AT3G53480.1	ABC transporter ABCG37	STLLDDGDESM_ox_TEKGR	88	DISP	INV		
AT3G63080.1	glutathione peroxidase	DSSGKEVDLSVYQGK	25	DISP	INV		yes
AT4G08620.1	SULTR1;1	DFKGQTPAK	55	DISP	INV		
AT4G33360.1	Farnesol deshydrogenase	NVLEAVKETKTVQK	112 or 115 or 119	DISP	INV		
AT4G37060.1	PATATIN-like protein 5	IDDDTLEGDASTLDLSTKSNLENLIK	340	DISP	INV		
AT5G14040.1	Mitochondrial phosphate carrier	FIKSEGYGGLYK	222	DISP	INV		
AT5G56010.1	HSP 90-3	APFDLFDTKK	326 or 327	DISP	INV		
AT5G65380.1	MATE efflux family protein	VANELGAGNGKGAR	334	DISP	INV		
**proteins with increased ubiquitination**							
AT1G01580.1	FRO2	DNDAGDEAKAGKIK	528 and 531	APP	INV		
AT1G02520.1	ABC transporter ABCB11	KQCEGPIKDGIK	919	APP	INV		
AT1G08930.2	ERD6	DTIDM_ox_TENGGETKMSELFQR	281	APP	INV		
AT1G08930.2	ERD6	DTIDM_ox_TENGGETKM_ox_SELFQR	281	APP	INV		
AT1G11680.1	Sterol 14-demethylase	SGKTENDM_ox_LQCFIESK	253	APP	INV		
AT1G12110.1	NRT1, NPF6.3	KLELPADPSYLYDVDDIIAAEGS_ph_M_ox_KGK	267 or 291 or 293	APP	INV		yes
AT1G44170.3	ALDH3H1	LSKLLDEK	242	APP	INV		
AT1G55450.1	methyl transferase	A_ac_ALSDKLADAYQNAR	6	2.3 (*p* = 0.01)	INV		
AT1G59870.1	ABC transporter ABCB11	EVDVTKLDGEDRQK	94	APP	INV		
AT1G61250.2	SCAMP1, secretory carrier3	ELQAKEAELK	71	APP	INV		yes
AT1G61670.1	Two-component response regulator	NELLFGLPDDVEEGKRE	511	APP	INV		
AT2G02040.1	NRT1, NPF 8.3	AAVISEEESKSGDYSNSWR	325	APP	INV	yes	yes
AT2G24720.1	glutamate receptor 2.2	DLWKEFLK	864	APP	INV		
AT2G32270.1	Zinc transporter 3	VSDGET_ph_GESSVDSEKVQILR	177	APP	INV		
AT2G38360.1	prenylated RAB acceptor	SALSKPESISDAAVR	68	1.72 (*p* = 0.02)	INV		
AT2G47000.1	ABC transporter ABCB4	A_ac_SESGLNGDPNILEEVSETKR	21	APP	INV		
AT3G04840.1	40S ribosomal protein S3a-1	IASEGLKHR	62	APP	INV		
AT3G08680.2	inactive receptor kinase	AYYFSKDEK	407 or 410	APP	INV	yes	
AT3G27770.2	hypoxia response protein	SPLIDGDNM_ox_VSFEKR	125	APP	INV	yes	yes
AT3G29310.1	BAG1	FVQYVDDCVVKR	230	APP	INV		
AT3G45710.1	NRT1, NPF2.5	DEDYHQYGLGKEAK	272	APP	INV		
AT3G51550.1	Feronia	AATKNFDESR	534	APP	INV		
AT3G53420.2	PIP2;1	ASGSKSLGS_ph_FR	276	1.79 (*p* = 0.01)	INV	yes *	
AT3G53420.2	PIP2;1	A_ac_KDVEAVPGEGFQTR	3	1.69 (*p* = 0.01)	INV	yes *	
AT3G60330.2	AHA7	TQHGLETGQKPVYER	903	APP	1.26 (*p* = 0.01)		
AT3G62250.1	40S ribosomal protein S27a-3	MQIFVKTLTGKTITLEVESSDTIDNVK	11	APP	INV		
AT4G01440.1	nodulin MtN21 EamA-like	FNEDDQEEDDDEQYKK	354 or 355	APP	INV		
AT4G09000.1	GRF1	AVDKDELTVEER	42	APP	INV		
AT4G25090.1	RBOHG	KELSDM_ox_LTESLKPTR	267	APP	INV		
AT4G30190.1	AHA2	AWLNLFENK	857	APP	1.44 (*p* = 0.01)	yes	yes
AT4G30190.1	AHA2	WSEQEAAILVPGDIVSIK	157	APP	1.44 (*p* = 0.01)	yes	yes
AT4G30190.1	AHA2	T_ph_LHGLQPKEAVNIFPEK	888	APP	1.44 (*p* = 0.01)	yes	yes
AT4G30190.1	AHA2	S_ac_SLEDIKNETVDLEK	8	3.00 (*p* = 0.02)	1.44 (*p* = 0.01)	yes	yes
AT5G25930.1	LRR protein kinase family	LLVYEYLEKR	767	APP	INV		
AT5G35200.1	clathrin assembly protein	EAPLAAGVKK	310	APP	INV		
AT5G39510.1	v-SNARE 11	KILTDM_ox_TR	184	APP	INV		
AT5G47910.1	RBOHD	NKLNLPNFLK	541	APP	INV		
AT5G59970.1	Histone superfamily protein	DNIQGITKPAIR	32	1.57 (*p* = 0.02)	INV		
AT5G62300.2	40S ribosomal protein S20-1	A_ac_TAYQPMKPGKAGLEEPLEQIHK	9 and 12	APP	INV		
AT5G62390.1	BAG7	AIAAAEAEKK	195	APP	INV		
AT5G62390.1	BAG7	LEPEYPLKYLCDR	90	3.02 (*p* = 0.03)	INV		
AT5G62390.1	BAG7	RLEPEYPLKYLCDR	90	2.09 (*p* = 0.01)	INV		

The table describes proteins for which protein and ubiquitin peptide quantification data are available. Columns 1 and 2: AGI and protein name. Column 3: the ubiquitinated residue is underlined. ox: oxidation; ac: acetylation; ph: phosphorylation. Column 4: K-Ub position in the protein. Column 5: the quantitative ratio of the ubiquitinated peptide between mannitol and control experiments, with the associated *p*-value. APP: appearance; DISP: disappearance. Column 6: the protein quantitative ratio between mannitol and control experiments, with the associated *p*-value. INV: invariant protein. Columns 7 and 8: protein interaction with UBC32 and UBC34 [15,16]. *: [17].

## Data Availability

The mass spectrometry proteomics data were deposited at the ProteomeXchange Consortium via the PRIDE partner repository with the dataset identifier PXD022249.

## Supplementary Materials

The following supporting information can be downloaded at: https://www.mdpi.com/article/10.3390/ijms23041956/s1.
