# Supplementary material for: Root Membrane Ubiquitinome under Short-Term Osmotic Stress
Source: Int J Mol Sci. 2022 Feb 10;23(4):1956. doi: 10.3390/ijms23041956 (PMC8879470; doi:10.3390/ijms23041956)
Supplement: Supplementary file 1 [file ijms-23-01956-s001.zip › supplemental Figures and Tables/Supplemental Figures S1-S9-revised.pdf]

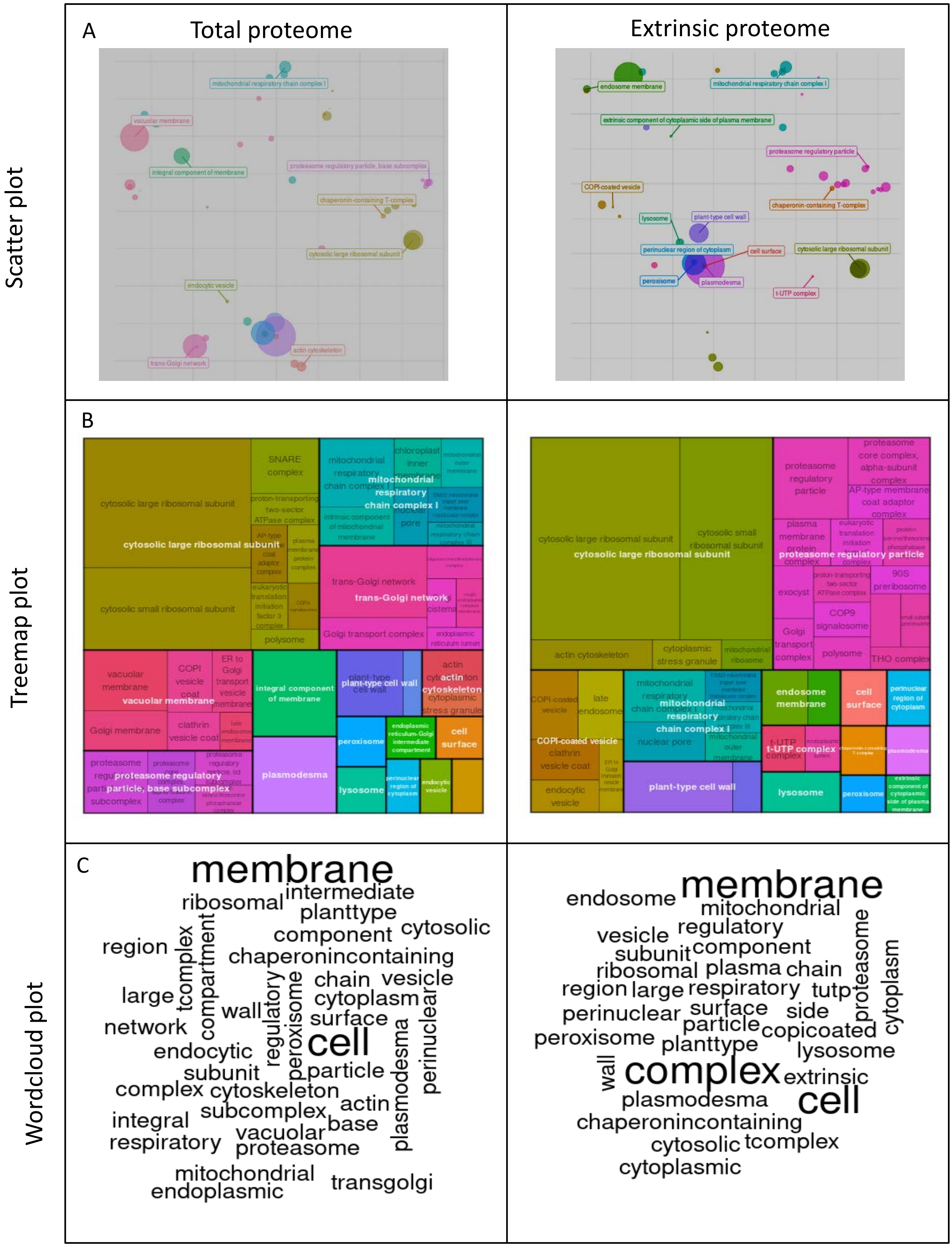

Figure S1. Characterization of the microsomal fraction. The functional enrichment analysis of cellular components of the total proteome and the extrinsic proteome (*i.e.* corresponding to proteins without transmembrane domains; Table S1) is shown. GO terms were reduced using rrvgo from the R package Bioconductor. Scatter (A), treemap (B), and wordcloud plots (C) are presented.

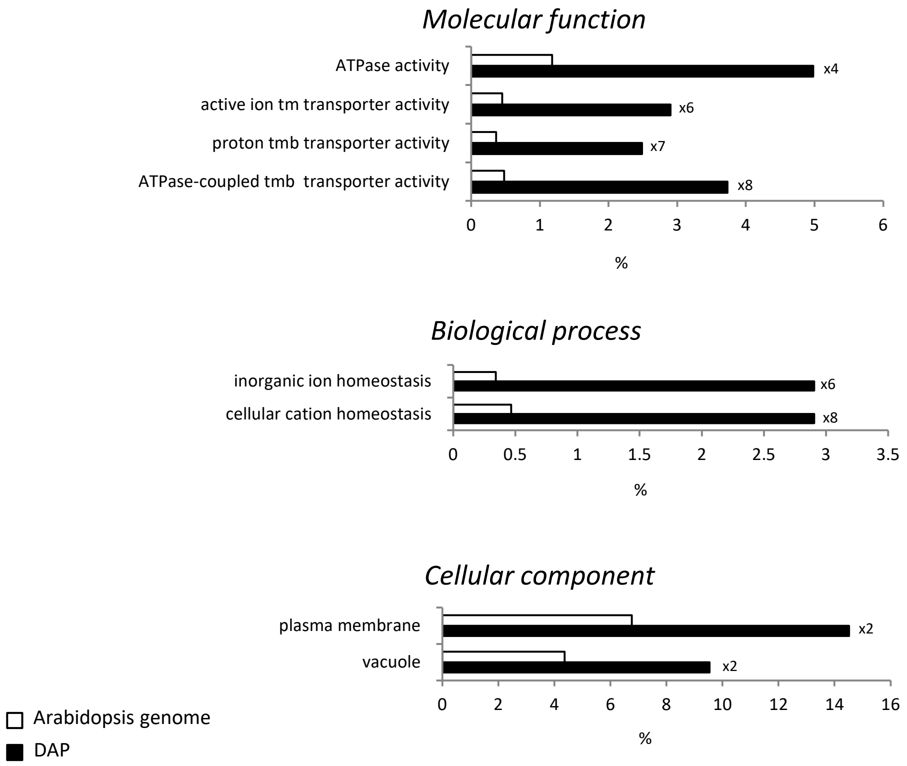

Figure S2. Functional enrichment analysis of differentially accumulated proteins (DAPs) in response to mannitol. The percentage is calculated with regard to the number of DAPs (black) and to the total number of Arabidopsis proteins (white). Numbers indicate the fold enrichment when compared to the Arabidopsis genome.

A

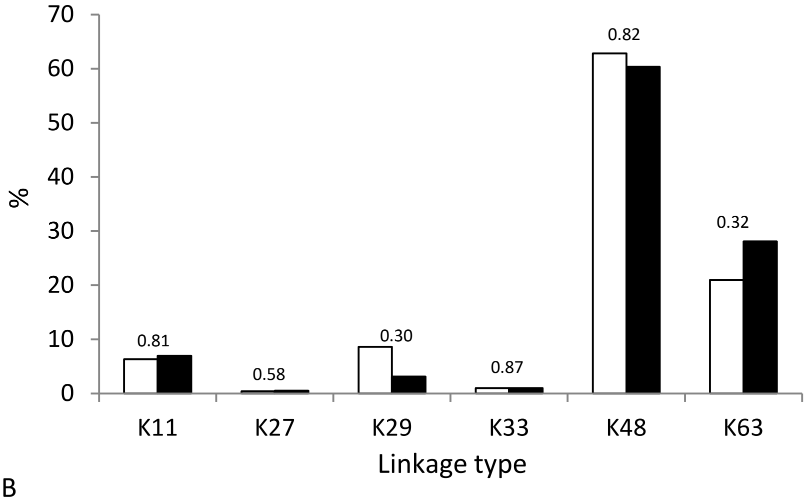

B

Peptide sequence	Modification	Ubiquitin linkage
TLTGKTITLEVESSDTIDNVK	GlyGly (K)	11
TLTGKTITLEVESSDTIDNVKAK	GlyGly (K)	11
TITLEVESSDTIDNVKAK	GlyGly (K)	27
AKIQDKEGIPPDQQR	GlyGly (K)	29
TITLEVESSDTIDNVKAKIQDK	Phospho (STY),GlyGly (K)	29
TITLEVESSDTIDNVKAKIQDK	GlyGly (K)	29
IQDKEGIPPDQQR	GlyGly (K)	33
LIFAGKQLEDGRTLADYNIQK	GlyGly (K)	48
LIFAGKQLEDGR	GlyGly (K)	48
LIFAGKQLEDGRTLADYNIQKESTLHLVLR	GlyGly (K)	48
QLEDGRTLADYNIQKESTLHLVLR	GlyGly (K)	63
TLADYNIQKESTLHLVLR	Phospho (STY),GlyGly (K)	63
TLADYNIQKESTLHLVLR	GlyGly (K)	63
TLADYNIQKESTLHLVLRLR	GlyGly (K)	63

Figure S3. Types of Ub linkages. (**A**) The proportion of Ub linkages in the control (white) and mannitol (black) ubiquitinomes. Numbers indicate p-values. (**B**) Ubi-peptides fitting with the different Ub linkages (see Table S4).

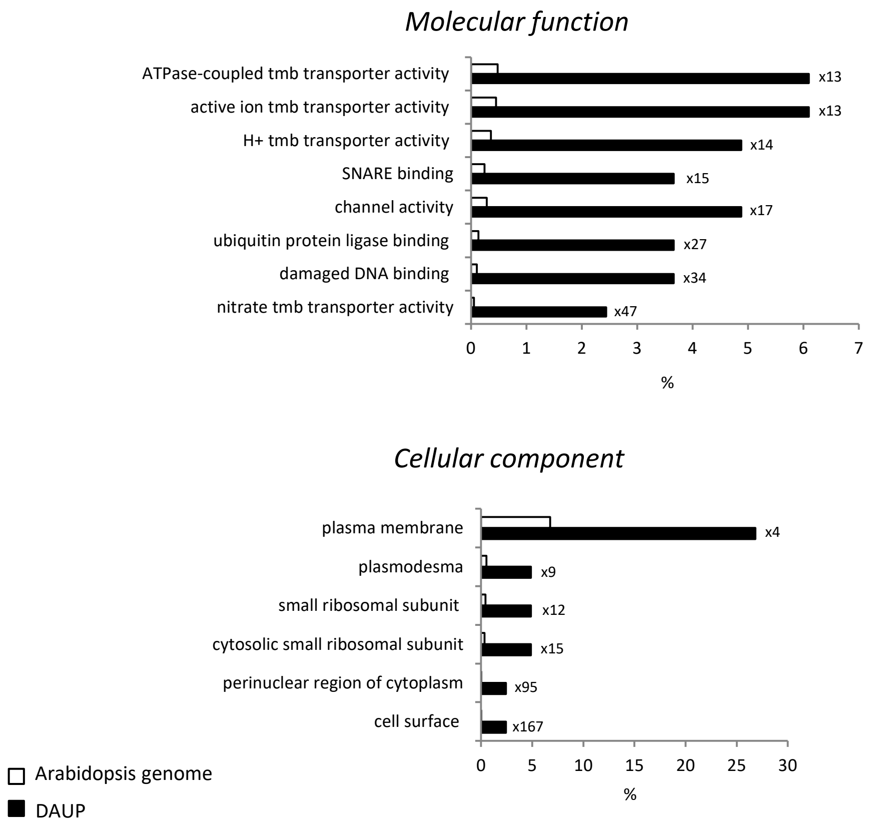

Figure S4. Functional enrichment analysis of differentially accumulated ubiquitinated proteins (DAUPs) in response to mannitol. The percentage is calculated with regard to the number of DAUPs (black) and to the total number of Arabidopsis proteins (white). Numbers indicate the fold enrichment when compared to the Arabidopsis genome. No enrichment in “biological process” was observed.

N-terminal alignment

```
PIP1;1      MEGKEEDVRVGANKFPERQPIGTSAQ-SDKDYKEPPPAPFFEPGELSSWSFWRAGIAEFI
PIP1;2      MEGKEEDVRVGANKFPERQPIGTSAQ-SDKDYKEPPPAPLFEPGELASWSFWRAGIAEFI
PIP1;3      MEGKEEDVRVGANKFPERQPIGTSAQ-TDKDYKEPPPAPFFEPGELSSWSFYRAGIAEFI
PIP1;4      MEGKEEDVRVGANKFPERQPIGTSAQSTDKDYKEPPPAPLFEPGELSSWSFYRAGIAEFI
PIP1;5      MEGKEEDVNVGANKFPERQPIGTAAQTESKDYKEPPPAPFFEPGELKSWSFYRAGIAEFI
PIP2;1      --------------MAKDVEAVPGEGFQTRDYQDPPPAPFIDGAELKKWSFYRAVIAEFV
PIP2;2      --------------MAKDVE--GPEGFQTRDYEDPPPTPFFDADELTKWSLYRAVIAEFV
PIP2;3      --------------MAKDVE--GPDGFQTRDYEDPPPTPFFDAEELTKWSLYRAVIAEFV
PIP2;4      --------------MAKDLDVNESGPPAARDYKDPPPAPFFDMEELRKWPLYRAVIAEFV
PIP2;5      --------------MTKEV-VGDKRSFSGKDYQDPPPEPLFDATELGKWSFYRALIAEFI
PIP2;6      --------------MTKDE-LTEEESLSGKDYLDPPPVKTFEVRELKKWSFYRAVIAEFI
PIP2;7      --------------MSKEV-SEEGKTHHGKDYVDPPPAPLLDMGELKSWSFYRALIAEFI
PIP2;8      --------------MSKEV-SEEG--RHGKDYVDPPPAPLLDMAELKLWSFYRAIIAEFI
```

C-terminal alignment

```
PIP1;1      YHVVVIRAIPFKSRS-------           286
PIP1;2      YHVIVIRAIPFKSRS-------           286
PIP1;3      YHQLVIRAIPFKSRS-------           286
PIP1;4      YHQIVIRAIPFKSKS-------           287
PIP1;5      YHQIVIRAIPFKSKT-------           287
PIP2;1      YHQFVLRASGSKSLGSFRSAANV----      287
PIP2;2      YHQFVLRASGSKSLGSFRSAANV----      285
PIP2;3      YHQFVLRASGSKSLGSFRSAANV----      285
PIP2;4      YHQFILRAAAIKALGSFGSFGSFRSFA      291
PIP2;5      INYHQFVLRAGAIKALGSFRSQPHV----    286
PIP2;6      YHQFVLRAGAMKAYGSVRSQLHELHA-      289
PIP2;7      YHQYILRASAIKALGSFRSNATN----      280
PIP2;8      YHQYILRAAAIKALASFRSNPTN----      278
```

Figure S5. K-Ub residues in PIP aquaporins. Red characters indicate identified ubiquitinated residues. Alignment was performed using the Clustal Omega program (https://www.ebi.ac.uk/Tools/msa/clustalo/).

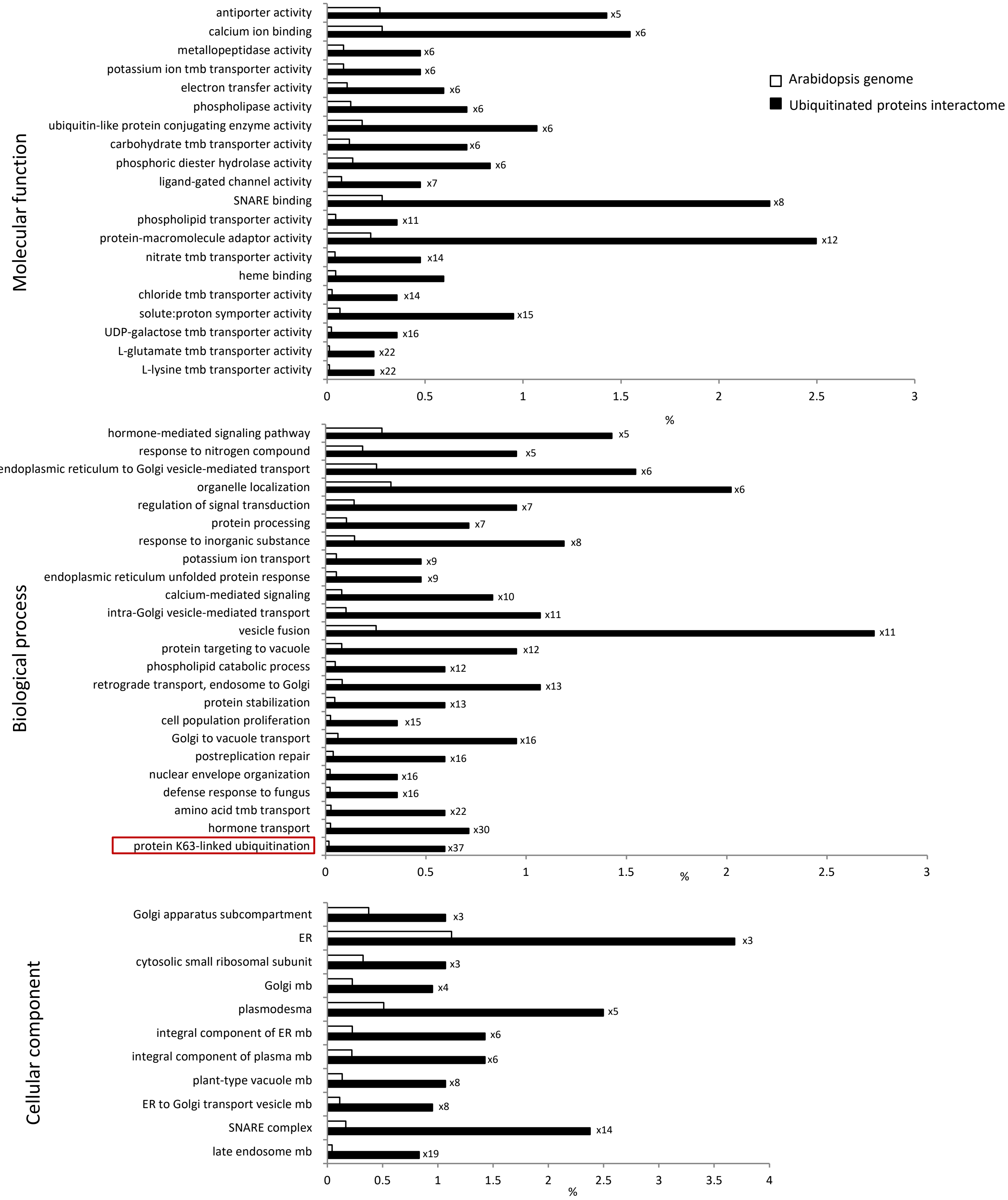

Figure S6. Functional enrichment analysis of the interactome of ubiquitinated proteins (corresponding to Figure 6). The percentage is calculated with regard to the number of proteins (black) and to the total number of Arabidopsis proteins (white). Numbers indicate the fold enrichment when compared to the Arabidopsis genome.
ER: endoplasmic reticulum. tmb: transmembrane.

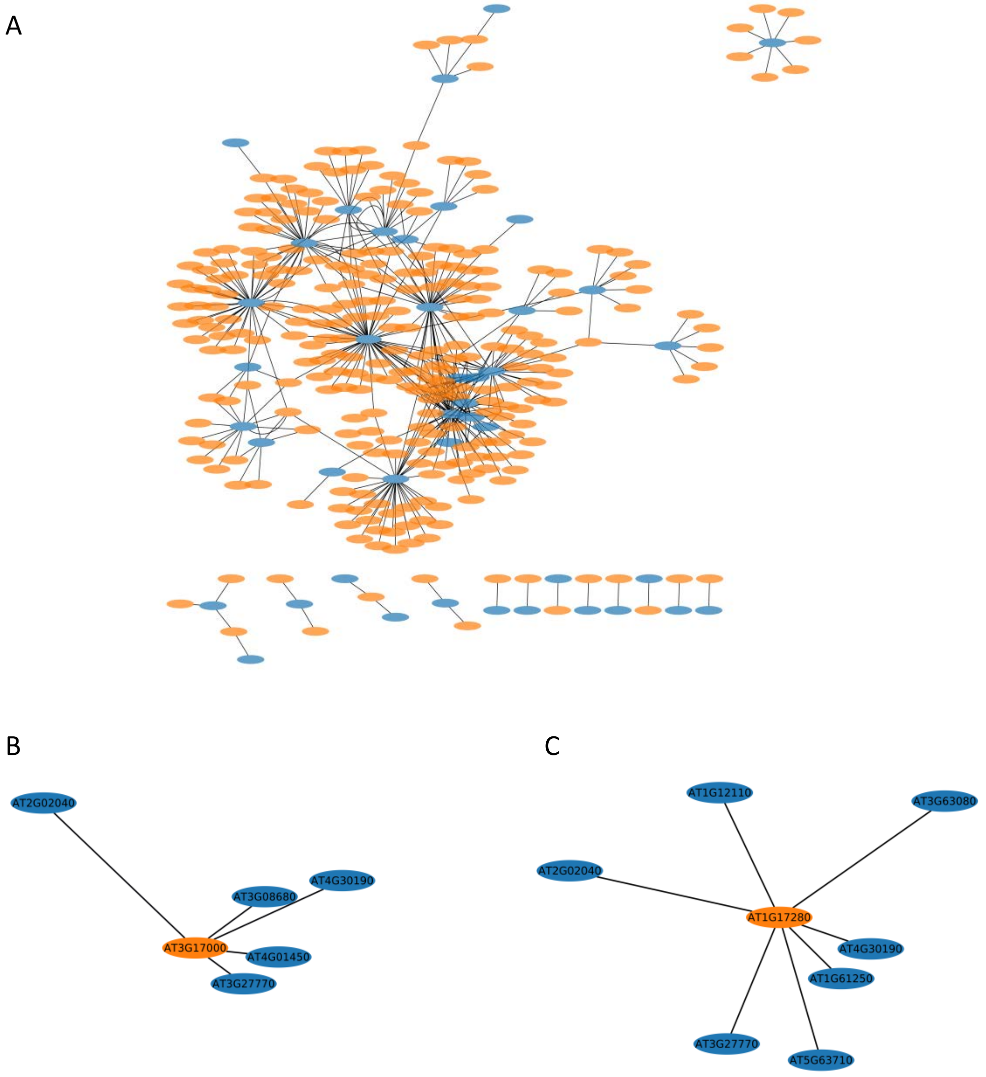

UBC32

AT2G02040	NRT1, PTR8.3
AT3G08680	LRR-LRK
AT3G27770	Unknown protein
AT4G01450	nodulin MtN21-like transporter
AT4G30190	AHA2

UBC34

AT1G12110	NRT1.1, PTR6.3
AT1G61250	SCAMP3
AT2G02040	NRT1, PTR8.3
AT3G27770	Unknown protein
AT3G63080	GPX5
AT5G63710	LRR-LRK
AT4G30190	AHA2

Figure S7. Interaction network of DAUPs. Interactants from a Y2H approach [15] and Split-Ub approach [16, 20] were considered, and the network was visualized by Cytoscape (version 3.7.2). A. The network includes DAUPs (blue) together with their reported interactants (orange) (Table S7d). B and C: E2s (UBC32 and UBC34) connecting DAUPs.

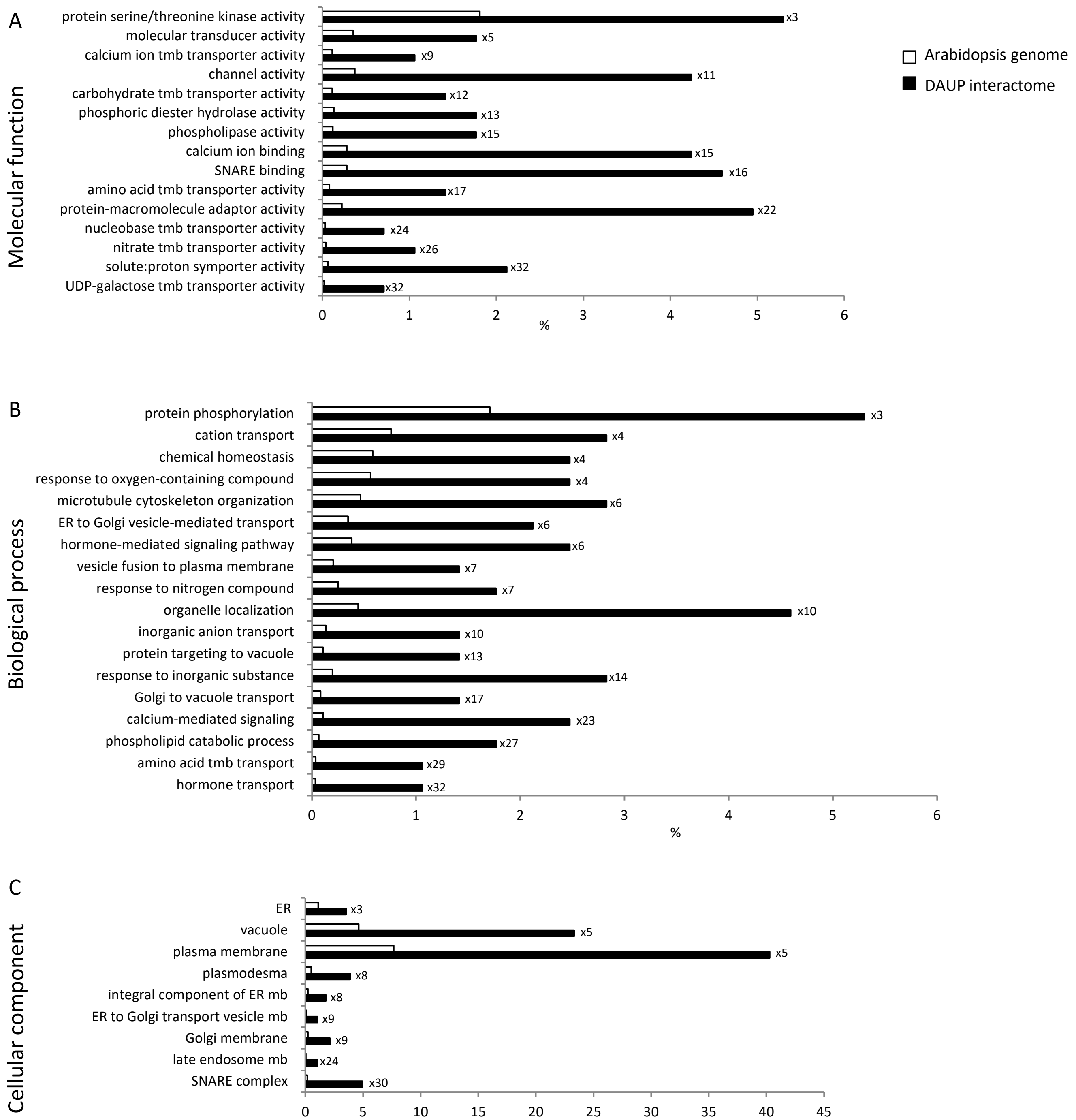

Figure S8. Functional enrichment analysis of the DAUP interactome. Proteins including DAUPs together with their reported interactants were considered. Functional enrichment analyses of molecular function (**A**), biological process (**B**), and cellular component (**C**) percentages were calculated with regard to the number of protein interactants (black) and the total number of Arabidopsis proteins (white). Numbers indicate the fold enrichment when compared to the Arabidopsis genome. No enrichment in “biological process” was observed.

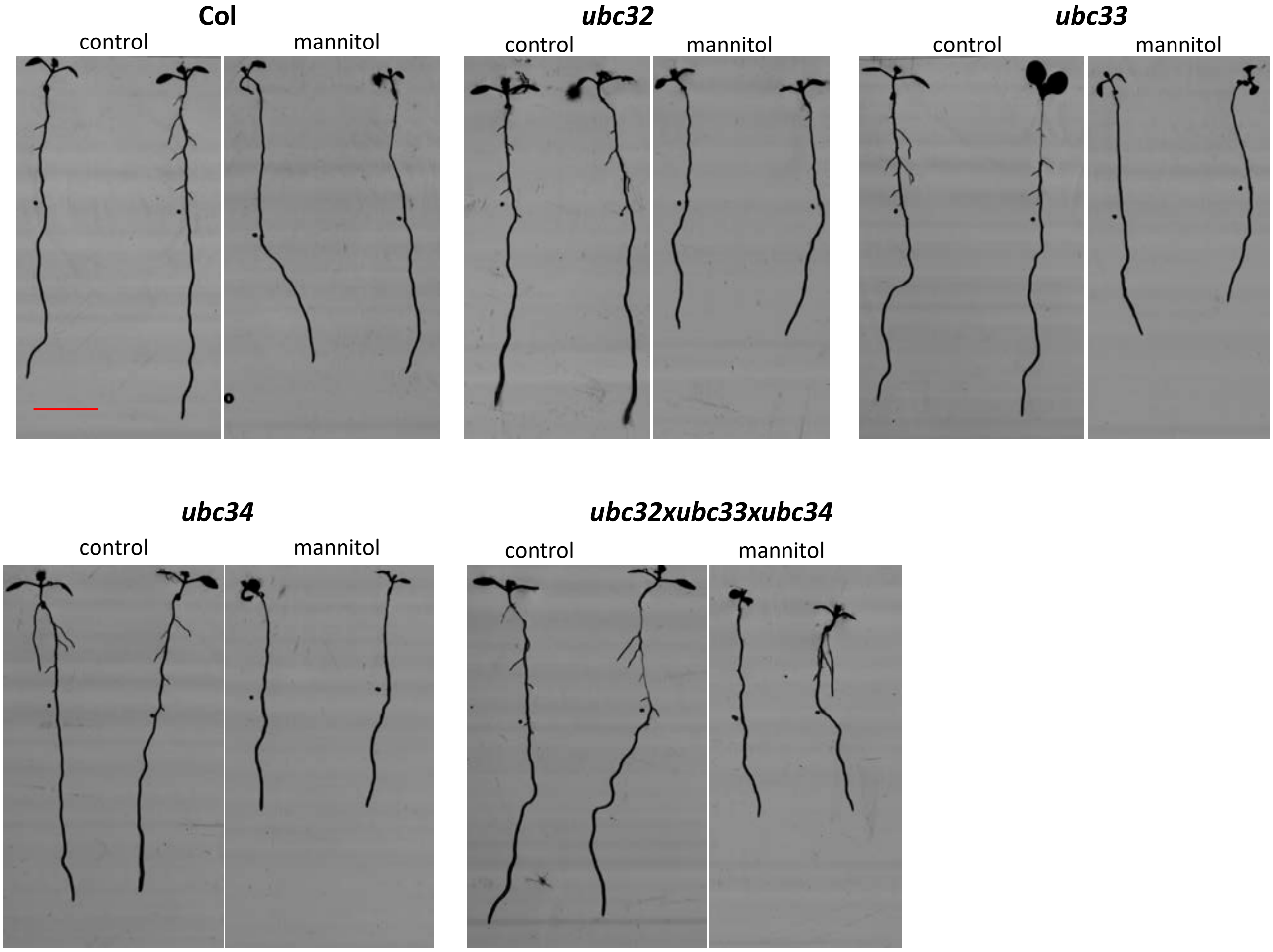

Figure S9. Root growth phenotype of WT plants and *ubc* mutants in 3-day control and 0.2 M mannitol conditions. The WT plant (Col), the *ubc32*, *ubc33*, *ubc34* mutants, and the triple mutant u*bc32xubc33xubc34* were grown for 5 days in control conditions and transferred for 3 days in MS/2 medium containing 0.2 M mannitol. Scale bar: 1 cm.
